# A Retrospective Comparative Study of the Effect of Controlled-Release Dinoprostone Vaginal Delivery System (Propess®) and Mechanical Methods for Cervical Ripening in Nulliparous Women in Late-Term Pregnancy

**DOI:** 10.7759/cureus.47255

**Published:** 2023-10-18

**Authors:** Yoshie Shibata, Nobuko Yokoyama, Shunji Suzuki

**Affiliations:** 1 Obstetrics and Gynecology, Japanese Red Cross Katsushika Maternity Hospital, Tokyo, JPN; 2 Obstetrics and Gynecology, Nippon Medical School, Tokyo, JPN

**Keywords:** propess, late-term, late-term pregnancy, nulliparous women, cervical ripening, mechanical methods, controlled-release dinoprostone vaginal delivery system (propess)

## Abstract

Objective: The effects of the controlled-release dinoprostone vaginal delivery system (Propess^®^) and mechanical methods for cervical ripening in nulliparous women in late-term pregnancy were compared retrospectively.

Methods: This retrospective comparative study included 46 nulliparous pregnant women (24 in the Propess^®^ group and 22 in the mechanical methods groups) with a low Bishop score (≤1) who needed labor induction at 41 weeks of gestation. The primary outcome was the success rate of cervical ripening (= Bishop score >6 or vaginal delivery) by the next day following the insertion of Propess^® ^only or mechanical cervical dilation only. In the cases in which cervical ripening was unsuccessful, other methods were performed, and the success rate of cervical ripening the day after was compared as the secondary outcome.

Results: As the primary outcome, there was not a significant difference in the success rate of cervical ripening between the Propess^® ^and mechanical methods groups (21 vs. 22%, p = 0.88). As for the secondary outcomes, there was not a significant difference in the total success rate of cervical ripening between the two groups (75 (5+13/24) vs. 73 (5+11/22)%, p = 0.86)). Of the unsuccessful cervical ripening cases as secondary outcomes, the Bishop score of all was ≤2 on the second day of hospitalization.

Conclusion: The combined use of Propess^® ^and mechanical methods was effective for cervical ripening in nulliparous women with a low Bishop score in late-term pregnancy, regardless of order.

## Introduction

Usually, pregnant women with an unfavorable cervix require cervical ripening prior to labor induction at term. In cases of a very low Bishop score (≤ 1) in nulliparous women, mechanical cervical dilation has been thought to be the safest and most effective method in Japan [1-4]. In 2020, however, controlled-release dinoprostone vaginal delivery systems (Propess®, Ferring Pharmaceuticals, Saint-Prex, Switzerland) were approved by the Ministry of Health, Labor, and Welfare of Japan [5]. Although Propess® seemed to be useful in the induction of labor in multiparous women with a high Bishop score before insertion at term [5-7], it cannot be denied that we may feel that the effect of cervical ripening is insufficient in nulliparous women with a significantly low Bishop score, regardless of the methods [8,9].

Based on the backgrounds, the objective of this study was to examine the success rate of cervical ripening of Propess® in nulliparous women with a low Bishop score requiring labor induction compared with that of mechanical methods.

## Materials and methods

The research was granted approval by the Ethics Committee of the Japanese Red Cross Katsushika Maternity Hospital, Tokyo, Japan, and all participants gave informed consent prior to their inclusion in this study.

This retrospective cohort study was conducted at our hospital, one of the main perinatal centers in Tokyo, Japan, between April 2020 and August 2023. In our institute, induction of labor is performed during the period of 41 weeks of gestation to prevent post-term pregnancy. Clinical data on material characteristics and obstetric outcomes were obtained from hospital records. The criteria for inclusion in this study were as follows: singleton pregnancy, cephalic presentation, gestational age at ≥41 weeks, and a Bishop score at the beginning of induction equal to or less than 1. The exclusion criteria were hypersensitivity to dinoprostone or its components in labor; maternal complications such as hypertensive disorder or gestational diabetes; previous uterine surgery; oligohydramnios; and fetal heart rate (FHR) abnormalities before intervention.

During this period, upon admission, internal examinations were performed to evaluate the Bishop score of each patient. When the Bishop Score ≤ 1 was observed, Propess® or mechanical methods (= insertion of laminaria tents or their synthetic equivalent, such as Dilapan in the cervical canal [3, 4]) were selected as a suitable intervention option freely. Successful cervical ripening was defined when the Bishop score was >6 or the patient had a vaginal delivery by the next day following the insertion of Propess® only or mechanical cervical dilation only as the primary outcome, while unsuccessful cervical ripening was defined when the Bishop score was ≤6. If the cervix is defined as ripening, oxytocin is usually used. In the unsuccessful cases, the other methods or the insertion of a catheter into the extra-amniotic space with balloon insufflation through the cervix (as an additional mechanical method) were performed [3,4], and the success rate of cervical ripening by the day after was compared as the secondary outcome.

Data are presented as numbers (percentages). Statistical analyses were performed with SAS version 8.02 (SAS Institute, Cary, NC, USA). For statistical analyses of categorical variables, the chi-square test or Fisher's exact test was used. Statistical significance was set at p<0.05.

## Results

Table 1 shows the clinical characteristics of the women in the Propess® and mechanical methods groups in this study; there were no significant differences in these variables between the two groups.

**Table 1:** Clinical characteristics of the women in the Propess® and mechanical methods groups Data are presented as numbers (percentage) BMI: body mass index

	Mechanical methods group	Propess^® ^group	p-value
Total	22	24	
Gestational age of 41 weeks	22 (100)	24 (100)	1
Maternal age ≥ 35 years	14 (64)	18 (75)	0.40
Maternal height < 150 cm	0 (0)	0 (0)	1
Maternal BMI at prepregnancy ≥ 25	3 (14)	4 (17)	0.78
Neonatal birth weight ≥ 3,500g	4 (18)	4 (17)	0.89

Table 2 shows the Bishop score and cervical ripening methods on the day of admission and the results on the next day in nulliparous women with a low Bishop score at 41 weeks of gestation.

**Table 2:** Cervical ripening methods on the day of admission and the results on the next day in nulliparous women with a low Bishop score at 41 weeks of gestation Data are presented as numbers (percentage)

	Mechanical methods group	Propess^® ^group	p-value
Total	22	24	
Successful cervical ripening			
Yes	5 (23)	5 (21)	0.88
No	17 (77)	18 (75)	-
Others (cesarean delivery)	0 (0)	1 (4)	1

As the primary outcome, there was not a significant difference in the success rate of cervical ripening between the Propess® and mechanical methods groups (21 vs. 23%, p=0.88), as shown in Table 2.

Table 3 shows the cervical ripening methods on the day after hospitalization and the results the next day in nulliparous women with a low Bishop score at 41 weeks of gestation.

**Table 3:** Cervical ripening methods on the day after hospitalization and the results the next day in nulliparous women with a low Bishop score at 41 weeks of gestation Data are presented as numbers (percentage)

	Mechanical methods group	Propess^® ^group	p-value
Total	17	18	
Bishop score			
0-2	11 (65)	13 (72)	
3-5	6 (35)	5 (28)	0.63
Cervical ripening methods			
Propess^®^	11 (65)		
Mechanical methods			
Laminaria tents or their synthetic equivalent	3 (18)	16 (89)	
Balloon insufflation	3 (18)	2 (11)	
Successful cervical ripening			
Yes	11 (65)	13 (72)	0.86
No	3 (18)	3 (17)	
Others (cesarean delivery)	3 (18)	2 (11)	0.66

As for the secondary outcomes, there was not a significant difference in the total success rate of cervical ripening between the two groups (75 (5+13/24) vs. 73 (5+11/22)%, p=0.86)).

There was not a significant difference in the total rate of vaginal delivery between the Propess® and mechanical methods groups (67 (16/24) vs. 73 (16/22)%, p=0.66)). Of the unsuccessful cervical ripening cases as secondary outcomes, the Bishop score of all was ≤2 on the second day of hospitalization.

## Discussion

In this study, there were not any significant differences in the success rate of cervical ripening between the Propess® and mechanical methods groups on the next and second days of hospitalization, regardless of order. In addition, the Bishop score in all unsuccessful cervical ripening cases as the secondary outcomes were ≤2 on the second day of hospitalization. These results indicated that the effects of Propess® and mechanical dilate do not differ regardless of the order in which they are used, and that if one is ineffective, the other may also be less effective.

In this study, the success rate of vaginal delivery was approximately 70%; however, considering that the average rate of cesarean sections in Japan [10,11], including multiparous women, is approximately 19%-25%, the current result is considered not bad at all. In addition, recently, the risk of a cesarean section was reported to be elevated with the use of Propess® in nulliparous women, especially in cases without epidural analgesia during labor [12]. Although we do not perform epidural anesthesia during the use of Propess® according to the Japanese guidelines [8], we were relieved that the use of Propess® did not lead to an increase in cesarean sections.

Based on the current results in nulliparous pregnant women in late-term pregnancy, although Propess® was not found to have the extreme effect of inducing labor that had previously been observed in multiparous women [6], Propess® was found to have a cervical ripening effect comparable to mechanical methods. The most favorable advantage of cervical ripening with Propess® is that it will be pain-free during insertion [13]. The success rate of cervical ripening with Propess® alone in this study was only 21%; however, the use of Propess® will be beneficial for nulliparous patients requiring induction of labor, as it has the same effect as mechanical methods without causing pain.

In this study, regardless of the order, the combination of the two methods was found to have a high success rate (approximately 75%) of cervical ripening in nulliparous women with a low Bishop score. Although there have been some reports comparing the effects of them in the past [4,13,14], this may be the first report to examine the effects of combining them. In addition, based on the current results, it was suggested that if one of the processes or mechanical methods is ineffective for cervical ripening, the other may also be ineffective. If neither of the two methods is effective, a less painful method may be more beneficial for pregnant women, including determining whether labor will proceed early [13]. Therefore, the benefits of using Propess® first may be estimated.

We understand that there are some limitations that cannot be ignored in this retrospective study. First of all, we cannot deny the small size of the cases in this study. In this study, we could not examine the incidence of uterine tachysystole associated with excessive uterine activity, which is a possible adverse event of prostaglandin E2 (PGE2) preparations [14,15]. However, there were not any cases of these complications associated with the small sample size in this study. In addition, while this study suggests that both methods are equally effective, the evidence presented may not strongly support this claim, and the results may be inconclusive. Therefore, further study with larger sample sizes and rigorous statistical analysis is necessary to provide more robust conclusions. Even when the Bishop score was consistent between the two groups, the possibility of bias at the discretion of the attending physician cannot be ruled out. In addition, although Propess® may have the potential benefit of being pain-free [13], we could not have any evaluation or discussion of pain levels or patient experience in comparison to mechanical methods. Therefore, a further prospective study may be required to consider the efficacy and risk of the two methods; however, a large price difference between the two methods (Propess® about $150 versus mechanical methods $15-75) may hinder further consideration. Otherwise, a discussion of cost-effectiveness may add depth to further study. The mention of a significant price difference between Propess® and mechanical methods will raise questions about the economic impact of these choices.

## Conclusions

In the nulliparous pregnant women with a low Bishop score at late-term, Propess® had a cervical ripening effect comparable to mechanical methods, and if one is ineffective, the other may also be less effective. In addition, the combined use of Propess® and mechanical methods was effective for cervical ripening in nulliparous women with a low Bishop score at late-term, regardless of order.

## References

[1] Chen W, Xue J, Peprah MK, Wen SW, Walker M, Gao Y, Tang Y (2016). A systematic review and network meta-analysis comparing the use of Foley catheters, misoprostol, and dinoprostone for cervical ripening in the induction of labour. BJOG.

[2] Warnock A (2017). Japan by the numbers: birth is too painful. http://www.tokyoreview.net/2017/08/japan-numbers-birth-painful-japan/.

[3] Maeda E, Ishihara O, Tomio J (2021). Cesarean delivery rates for overall and multiple pregnancies in Japan: a descriptive study using nationwide health insurance claims data. J Obstet Gynaecol Res.

[4] Yokoyama N, Suzuki S (2023). Comparison of obstetric outcomes between controlled-release dinoprostone vaginal delivery system (PROPESS) and administration of oral dinoprostone for labor induction in multiparous women at term. Cureus.

[5] Itakura A, Shoji S, Shigeru A (2023). Guidelines for obstetrical practice in Japan: Japan Society of Obstetrics and Gynecology and Japan Association of Obstetricians and Gynecologists 2020 edition. J Obstet Gynaecol Res.

[6] Levine LD (2020). Cervical ripening: why we do what we do. Semin Perinatol.

[7] de Vaan MD, Ten Eikelder ML, Jozwiak M (2019). Mechanical methods for induction of labour. Cochrane Database Syst Rev.

[8] Itoh H, Ishii K, Shigeta N (2021). Efficacy and safety of controlled-release dinoprostone vaginal delivery system (PROPESS) in Japanese pregnant women requiring cervical ripening: results from a multicenter, randomized, double-blind, placebo-controlled phase III study. J Obstet Gynaecol Res.

[9] Ezebialu IU, Eke AC, Eleje GU, Nwachukwu CE (2015). Methods for assessing pre-induction cervical ripening. Cochrane Database Syst Rev.

[10] LeFevre NM, Krumm E, Cobb WJ (2021). Labor dystocia in nulliparous women. Am Fam Physician.

[11] Grobman WA, Bailit J, Lai Y (2017). Defining failed induction of labor. Am J Obstet Gynecol.

[12] Kim YM, Park JY, Sung JH, Choi SJ, Oh SY, Roh CR, Kim JH (2017). Predicting factors for success of vaginal delivery in preterm induction with prostaglandin E2. Obstet Gynecol Sci.

[13] Anh ND, Duc TA, Ha NT (2022). Dinoprostone vaginal insert for induction of labor in women with low-risk pregnancies: a prospective study. Med Arch.

[14] Druenne J, Semay T, Giraud A, Chauleur C, Raia-Barjat T (2022). Pain and satisfaction in women induced by vaginal dinoprostone, double balloon catheter and oral misoprostol. J Gynecol Obstet Hum Reprod.

[15] Jones MN, Palmer KR, Pathirana MM (2022). Balloon catheters versus vaginal prostaglandins for labour induction (CPI Collaborative): an individual participant data meta-analysis of randomised controlled trials. Lancet.

